# Yield of bone scintigraphy screening for transthyretin‐related cardiac amyloidosis in different conditions: Methodological issues and clinical implications

**DOI:** 10.1111/eci.13665

**Published:** 2021-08-22

**Authors:** Giacomo Tini, Eugenio Sessarego, Stefano Benenati, Pier Filippo Vianello, Beatrice Musumeci, Camillo Autore, Marco Canepa

**Affiliations:** ^1^ Cardiology Unit Department of Internal Medicine University of Genoa Genoa Italy; ^2^ Division of Cardiology Sapienza University of Rome Sant’Andrea Hospital Rome Italy; ^3^ Cardiology Unit Ospedale Policlinico San Martino IRCCS Genoa Italy

**Keywords:** aortic stenosis, carpal tunnel syndrome, heart failure, left ventricular hypertrophy, transthyretin cardiac amyloidosis

## Abstract

**Background:**

Transthyretin‐related cardiac amyloidosis (TTR‐CA) is thought to be particularly common in specific at‐risk conditions, including aortic stenosis (AS), heart failure with preserved ejection fraction (HFpEF), carpal tunnel syndrome (CTS) and left ventricular hypertrophy or hypertrophic cardiomyopathy (LVH/HCM).

**Methods:**

We performed a systematic revision of the literature, including only prospective studies performing TTR‐CA screening with bone scintigraphy in the above‐mentioned conditions. Assessment of other forms of CA was also evaluated. For selected items, pooled estimates of proportions or means were obtained using a meta‐analytic approach.

**Results:**

Nine studies (3 AS, 2 HFpEF, 2 CTS and 2 LVH/HCM) accounting for 1375 screened patients were included. One hundred fifty‐six (11.3%) TTR‐CA patients were identified (11.4% in AS, 14.8% in HFpEF, 2.6% in CTS and 12.9% in LVH/HCM). Exclusion of other forms of CA and use of genetic testing was overall puzzled. Age at TTR‐CA recognition was significantly older than that of the overall screened population in AS (86 vs. 83 years, *p* = .04), LVH/HCM (75 vs. 63, *p* < .01) and CTS (82 vs. 71), but not in HFpEF (83 vs. 79, *p* = .35). In terms of comorbidities, hypertension, diabetes and atrial fibrillation were highly prevalent in TTR‐CA‐diagnosed patients, as well as in those with an implanted pacemaker.

**Conclusions:**

Screening with bone scintigraphy found an 11–15% TTR‐CA prevalence in patients with AS, HFpEF and LVH/HCM. AS and HFpEF patients were typically older than 80 years at TTR‐CA diagnosis and frequently accompanied by comorbidities. Several studies showed limitations in the application of recommended TTR‐CA diagnostic algorithm, which should be addressed in future prospective studies.

## 1 INTRODUCTION

There is growing clinical awareness regarding transthyretin (TTR)‐related cardiac amyloidosis (CA), partly driven by the surfacing of specific disease‐modifying treatments both for variant (TTRv) and for wild‐type (TTRwt) TTR‐CA.^1^, ^2^ Moreover, the disease is increasingly recognized due to the fact that nonbiopsy diagnosis with bone scintigraphy is now possible in many cases.^3^ The epidemiology of TTR‐CA has thus changed in recent years.^4^

TTR‐CA is thought to be particularly common in specific subsets of patients,^2^, ^5^, ^6^, ^7^ including those with aortic stenosis (AS), heart failure with preserved ejection fraction (HFpEF), carpal tunnel syndrome (CTS) and left ventricular hypertrophy or hypertrophic cardiomyopathy (LVH/HCM), especially when diagnosed in adults. Screening for TTR‐CA in these populations at risk was reported to lead to a consistent number of diagnosed cases. Nevertheless, a systematic and critical assessment of contemporary screening strategies for TTR‐CA in these different conditions is lacking to date.

The aim of this work was to comprehensively investigate results from prior prospective studies in which screening for TTR‐CA was performed by bone scintigraphy in these populations at risk.

## 2 METHODS

**FIGURE 1:**
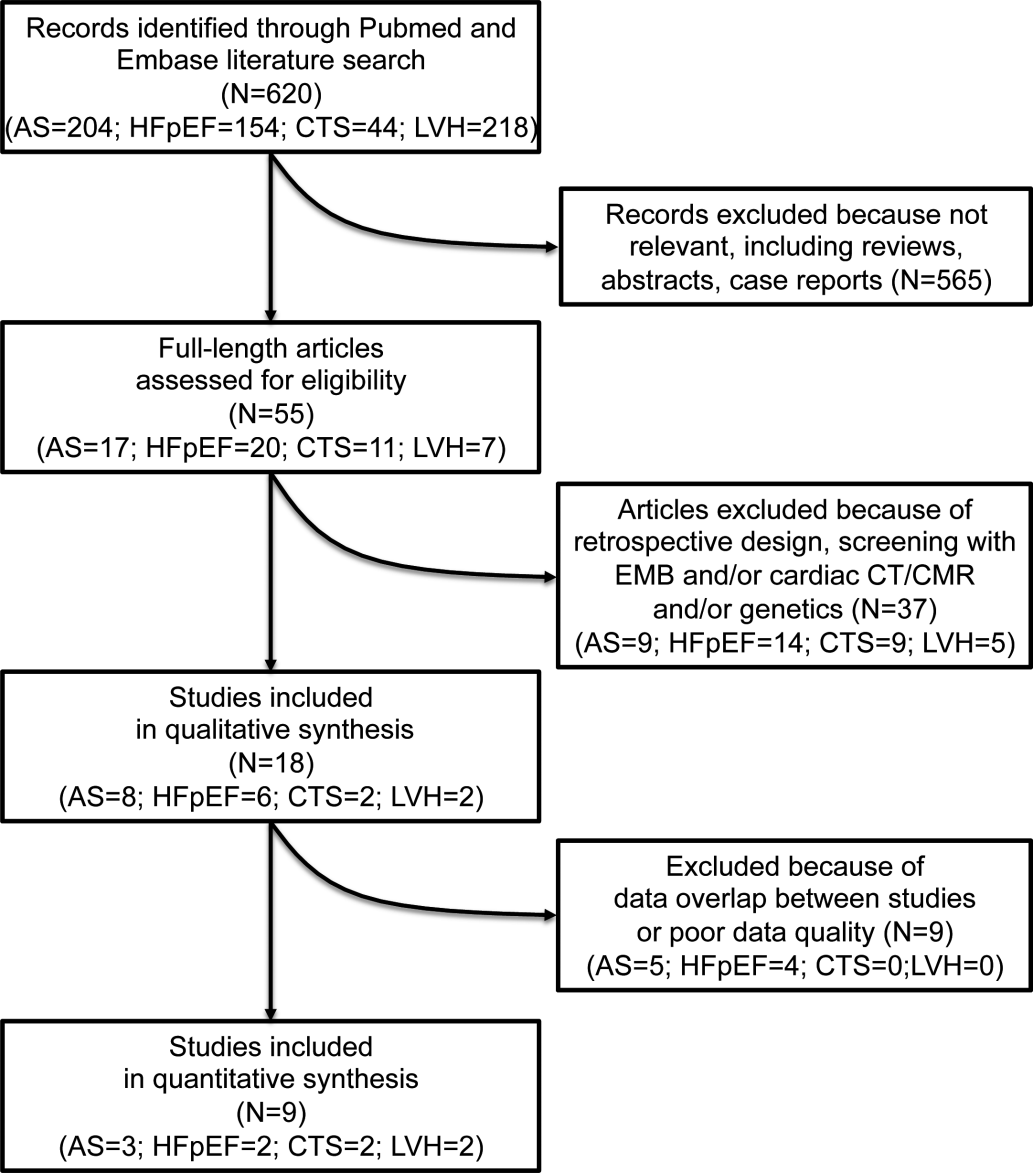
Flow diagram of studies’ screening and selection process. AS, aortic stenosis; CMR, cardiac magnetic resonance; CT, computerized tomography; CTS, carpal tunnel syndrome; EMB, endomyocardial biopsy; HFpEF, heart failure with preserved ejection fraction; LVH, left ventricular hypertrophy. See Supplementary Material for further details

We performed a systematic revision of the literature in PubMed/Embase to identify prospective studies conducting screening for TTR‐CA by the means of bone scintigraphy in each of the following at‐risk conditions: AS undergoing replacement; HFpEF; CTS; and LVH/HCM. The search is updated to December 2020. Reviews and retrospective studies, as well as those performing screening by means of genetics or cardiac magnetic resonance or endomyocardial biopsy or intraoperative biopsy or autopsy—without prior or concomitant bone scintigraphy—were excluded (Figure 1). Moreover, studies with a consistent number of missing variables of interest (i.e., detailed information regarding comorbidities such as hypertension, diabetes and atrial fibrillation, or implanted devices) or that enrolled less than 10 patients were excluded. Publications deemed as having considerable overlap with one another were carefully reviewed, and only the major one was retained in the final analysis. Details regarding systematic revision and exclusion criteria are reported in the Supplementary Material, according to the PRISMA (Preferred Reporting Items for Systematic Reviews and Meta‐Analyses) methodology.^8^ Reporting of the study conforms to broad EQUATOR guidelines.^9^ Available means/medians and frequencies obtained or derived from the original manuscripts were averaged when the same variable from two or more samples within each condition was available. Because of the limited numbers and heterogeneity of included studies, further results were mostly derived from analyses of descriptive statistics with no formal statistical testing. Using a meta‐analytic approach, pooled estimates of proportions (in case of binomial variables) and retrievable means (in case of continuous variables) were obtained for selected items, and differences between subgroups were tested. Results of random‐effect models were reported in case of intermediate‐to‐high heterogeneity, as defined by an *I* ^2^ ≥ 25%.^10^ Contrariwise, results of fixed‐effect models were chosen in case of low heterogeneity (*I* ^2^ < 25%). The analysis was conducted using R (The R Foundation for statistical computing, Wien).

## 3 RESULTS

### 3.1 Methodology of included studies

Nine studies, accounting for 1375 screened patients, were included in the present analysis: 3 for AS,^11^, ^12^, ^13^ 2 for HFpEF,^14^, ^15^ 2 for CTS^16^, ^17^ and 2 for LVH/HCM^18^, ^19^ (Table 1). Included studies’ year of publication ranged from 2015 to 2020.

**TABLE 1:** Major characteristics of studies included in the analysis

	Aortic stenosis undergoing replacement				HFpEF			Carpal tunnel syndrome			LVH/HCM		
	Nitsche^11^ et al*	Rosemblum^12^ et al	Scully^13^ et al	Total	Benanni Smires^14^ et al	Gonzalez‐Lopez^15^ et al	Total	Vianello^16^ et al	Zegri‐Reiriz^17^ et al	Total	Maurizi^18^ et al**	Cariou^19^ et al*	Total
Scintigraphic bone tracer													
	^99m^Tc‐DPD	^99m^Tc‐PYP	^99m^Tc‐DPD		^99m^Tc‐DPD	^99m^Tc‐DPD		^99m^Tc‐HMDP	^99m^Tc‐DPD		/	^99m^Tc‐HMDP	
Number of patients													
All	191	204	200	595	49	120	169	53	101	154	343	114	457
TTRwt‐CA	15	27	26	68	9	16	25	2	2	4	17	26	43
TTRv‐CA	0	/	/	/	0	0	0	0	0	0	11	5	16
Other CA	1	0	0	1	6	0	6	0	1	1	4	19	23
***TTR‐CA prevalence***	*7.9%*	*13.2%*	*13.0%*	***11.4%***	*18.4%*	*13.3%*	***14.8%***	*3.8%*	*1.9%*	***2.6%***	*8.2%*	*27.2%*	***12.9%***
Age, years (Mean/median)													
All	82 (78–86)	83 ± 7	85 ± 5	83	76 ± 8	82 ± 8	79	72 (42–95)	69 (64–77)	71	60 ± 13	65 ± 21	63
TTR‐CA	84 (81–89)	86 ± 5	88 ± 5	86	80 ± 5	86 ± 6	83	79 ± 0.5	85.5 ± 0.7	82	77 ± 6	72 ± 12*	75
Male pts, *n* (%)													
All	95 (49.7)	133 (65.2)	99 (49.5)	327 (54.9)	28 (57.1)	49 (40.8)	77 (45.6)	53 (100.0)	32 (31.6)	85 (55.2)	199 (58.0)	84 (73.7)	283 (61.9)
TTR‐CA	10 (62.5)	26 (96.3)	16 (61.5)	52 (76.5)	9 (100.0)	8 (50.0)	17 (68.0)	2 (100.0)	0 (0.0)	2 (50.0)	15 (88.2)	/(80.0)*	/(84.1)
Hypertension, *n* (%)													
All	167 (87.3)	175 (85.7)	154 (77)	496 (83.4)	34 (69.4)	101 (84.1)	135 (79.8)	30 (56.6)	65 (64.4)	95 (61.7)	/	69 (60.5)	/
TTR‐CA	14 (87.5)	25 (92.6)	19 (73.1)	58 (85.3)	2 (22.2)	14 (87.5)	16 (64.0)	/	/	/	/	/ (44.0)*	/
Diabetes, *n* (%)													
All	50 (26.2)	/	48 (24.0)	98 (25.1)	13 (26.5)	45 (37.5)	58 (34.3)	6 (11.3)	32 (31.6)	38 (24.7)	/	22 (19.3)	/
TTR‐CA	5 (31.2)	/	3 (11.5)	8 (21.4)	2 (22.2)	4 (25.0)	6 (24.0)	/	/	/	/	/(16.0)*	/
Atrial fibrillation, *n* (%)													
All	72 (37.7)	83 (40.7)	74 (37.0)	229 (38.5)	33 (67.3)	80 (66.7)	113 (66.9)	7 (13.2)	7 (6.9)	14 (9.1)	75 (21.8)	41 (35.9)	116 (25.4)
TTR‐CA	9 (56.3)	10 (37.0)	11 (42.3)	30 (44.1)	8 (88.8)	13 (81.2)	21 (84.0)	1 (50.0)	0 (0.0)	1 (25.0)	4 (23.5)	/(50.0)*	/(36.8)
Pacemaker, *n* (%)													
All	25 (13.1)	/	23 (11.5)	48 (12.3)	0 (0.0)	19 (15.8)	19 (11.2)	2 (3.8)	5 (4.9)	7 (4.5)	13 (3.8)	25 (21.9)	38 (8.3)
TTR‐CA	5 (31.2)	/	4 (15.3)	9 (23.2)	0 (0.0)	7 (43.8)	7 (28.0)	0 (0.0)	0 (0.0)	0 (0.0)	1 (5.8)	/(34.0)*	/(19.9)
NT‐proBNP, ng/L (Mean/median)													
All	1917 (783–5893)	2142 (1002–5712)	1467 (640–3337)	1842	/	3524 (1500–7500)	/	/	150 (57–316)	/	/	/	/
TTR‐CA	3634 (1241 –6323)	3132 (1812–6138)	3702 (1286–5626)	3489	2868 ± 2822	6467 (2818–13146)	4668	/	2045 ± 1701	/	7276 ± 6344	3278*	/

Note

Values are reported as mean ± SD or median and interquartile ranges according to the original study publication. For studies reporting BNP values, these were multiplied by 6 to obtain corresponding NT‐proBNP values.

*This study presented data aggregated for all CA cases (ATTRwt, ATTRv and other CA, including AL). However, the study by Nitsche et al^11^ had only 1 of 16 case of non‐TTR‐CA, whereas the study by Cariou et al^19^ had 19 of 50 cases of non‐TTR‐CA. Thus, for this study only percentages were presented and averaged.

**For this study, only data for ATTRwt‐CA are presented, given that this was the only condition screened with bone scintigraphy.

99m Tc‐DPD, ^99m^Tc‐3,3‐diphosphono‐1,2‐propanodicarboxylic acid; ^99m^Tc‐PYP, ^99m^Tc‐pyrophosphate; ^99m^Tc‐HMDP, ^99m^Tc‐hydroxymethylene‐diphosphonate; HFpEF, heart failure with preserved ejection fraction; LVH, left ventricular hypertrophy; HCM, hypertrophic cardiomyopathy.

^99m^Tc‐3,3‐diphosphono‐1,2‐propanodicarboxylic acid (^99m^Tc‐DPD) was the scintigraphic bone tracer more commonly used (in 5 studies). Two AS studies^11^, ^12^ and one HFpEF study^15^ investigated only patients with a moderate‐to‐severe scintigraphic uptake (i.e., Perugini score ≥2). In one AS study,^13^ a plasma cell dyscrasia was detected in 6 patients with a positive bone scan; however, these patients did not undergo endomyocardial biopsy as, according to detailed clinical revisions, light‐chain CA ‘was felt unlikely’. In one HFpEF study,^15^ serum‐free light‐chain assay and serum and urine immunofixation were not performed. Patients with positive bone scintigraphy underwent extracardiac or cardiac biopsy ‘if considered by the treating physician’. Finally, in one LVH/HCM study,^19^ light‐chain CA presence was ascertained only in patients with negative bone scintigraphy.

### 3.2 Prevalence of TTR‐CA and characteristics of patients

Overall, 156 (11.3%) TTR‐CA patients were identified by means of screening with bone scintigraphy. The prevalence of TTR‐CA varied between settings: 11.4% in AS, 14.8% in HFpEF, 2.6% in CTS and 12.9% in LVH/HCM. Out of the 156 TTR‐CA identified patients, 140 (89.7%) were TTRwt‐CA and 16 (10.3%) were TTRv‐CA. All TTRv‐CA patients were identified in the LVH/HCM setting. Recognition of other CA aetiologies was low, with a total of 31 (2.3%) patients, mostly identified in the LVH/HCM setting (23 out of 31 patients).

Table 1 shows clinical characteristics of all patients enrolled and of TTR‐CA patients identified in each study. The gender of screened populations was roughly half male and half female; a male predominance was present in patients diagnosed with TTR‐CA in the AS and LVH/HCM settings, and less marked in the HFpEF setting. Although one CTS study involved only male patients,^16^ in this setting, out of the 4 TTR‐CA patients identified, 2 were males and 2 females. Age of screened populations was similar in AS and HFpEF (83 vs. 79 years, *p *= .12), but significantly greater in AS than in CTS (71 years) or LVH/HCM (63 years, *p *< .01 for both). In AS studies, there were no inclusion/exclusion age cut‐offs, which were instead set in both HFpEF studies (≥60 and 65 years),^14^, ^15^ in one CTS study (≥60 years)^17^ and in one LVH/HCM study (≥40 years).^18^ Age at TTR‐CA recognition was significantly older than that of the overall screened population in AS (86 vs. 83 years, *p *= .04), LVH/HCM (75 vs. 63, *p *< .01) and CTS (71 vs. 82, *p* not calculable), but not in HFpEF (83 vs. 79, *p *= 0.35).

In terms of comorbidities, AS and HFpEF screened populations were more frequently affected by arterial hypertension as compared to CTS and LVH/HCM patients (about 81.6% vs. about 61.1%, respectively, *p *< .01 for all). The presence of arterial hypertension was comparable in patients with and without TTR‐CA within each condition. About one fourth of AS and HFpEF screened patients had diabetes, with a similar rate in those with and without TTR‐CA. Atrial fibrillation appeared relatively more common, within each condition, in patients with TTR‐CA than in the screened population (AS: 44.1 vs. 38.5%, *p *= .30; HFpEF: 84.0 vs. 66.9%, *p *= .07; CTS: 25.0 vs. 9.1%, *p *= .31; LVH/HCM: 36.8 vs. 25.4%, *p *= .25), and significantly more frequent in TTR‐CA HFpEF patients than in TTR‐CA associated with other conditions (*p *< .03 for all comparisons). Finally, about 10% of screened patients within each condition had a pacemaker already implanted; this percentage doubled or tripled in patients diagnosed with TTR‐CA (AS: 23.2 vs. 12.3%; HFpEF: 28.0 vs. 11.2%; LVH/HCM: 19.9 vs. 8.3%, respectively), but not in those with CTS (4.5 vs. 0.0%).

Outcomes were assessed only in AS studies, which reported no differences in terms of mortality between AS patients with and without CA.^11^, ^12^, ^13^ One study^12^ found a significantly higher rate of heart failure hospitalizations in AS‐CA patients at 1 years after valve replacement, but not at 3 years.

## 4 DISCUSSION

We herein present a systematic revision of prospective studies that performed bone scintigraphy screening for TTR‐CA in different populations at risk. Our main findings pertain the methodology of these studies and the epidemiological scenario that they depict.

### 4.1 Methodological considerations

Although conditions deemed at risk of TTR‐CA have been long and widely identified,^5^ few are the prospective screening studies that used a contemporary validated workup including bone scintigraphy, according to the Gillmore algorithm.^3^ About half of the 9 studies included in this analysis had inherent limitations regarding the diagnostic approach to TTR‐CA. Three studies^13^, ^15^, ^19^ did not perform a thorough exclusion of light‐chain CA; three studies^11^, ^12^, ^15^ investigated only patients with a moderate‐to‐severe scintigraphic uptake. This approach is certainly more specific and less time‐consuming, as Perugini 1 patients should always undergo biopsy confirmation also in the absence of plasma cell dyscrasia,^2^, ^3^, ^20^ but may have caused some CA diagnoses to be missed (especially non‐TTR‐CA cases). It is important to underline that the two included HFpEF studies were published before the 2016 publication by Gillmore and colleagues.^3^

The two HFpEF and CTS studies had different inclusion criteria and may have not been completely comparable: Bennani Smires and colleagues^14^ enrolled HFpEF patients older than 65 years, while González‐Lopez and colleagues^15^ enrolled HFpEF patients older than 60 years and with LVH; Vianello and colleagues^16^ enrolled only male patients with bilateral CTS, while Zegri‐Reiriz and colleagues^17^ enrolled both males and females aged ≥60 years with monolateral or bilateral CTS.

Finally, genetic TTR testing was not routinely performed in two AS study,^12^, ^13^ whereas in all other studies, it was generally done in patients with a positive bone scintigraphy. Nevertheless, it is known that some mutations may not lead to positive bone scintigraphy even in the presence of TTRv‐CA.^2^, ^21^ This possibility should be at least taken into account in particular for two studies^14^, ^19^ in which a subgroup of patients had negative bone scintigraphy, but a definitive diagnosis was not reached (i.e., ‘unspecified’). Altogether, these limitations may have partly hindered the real prevalence of TTR‐CA across the different conditions.

### 4.2 Epidemiological considerations

**FIGURE 2:**
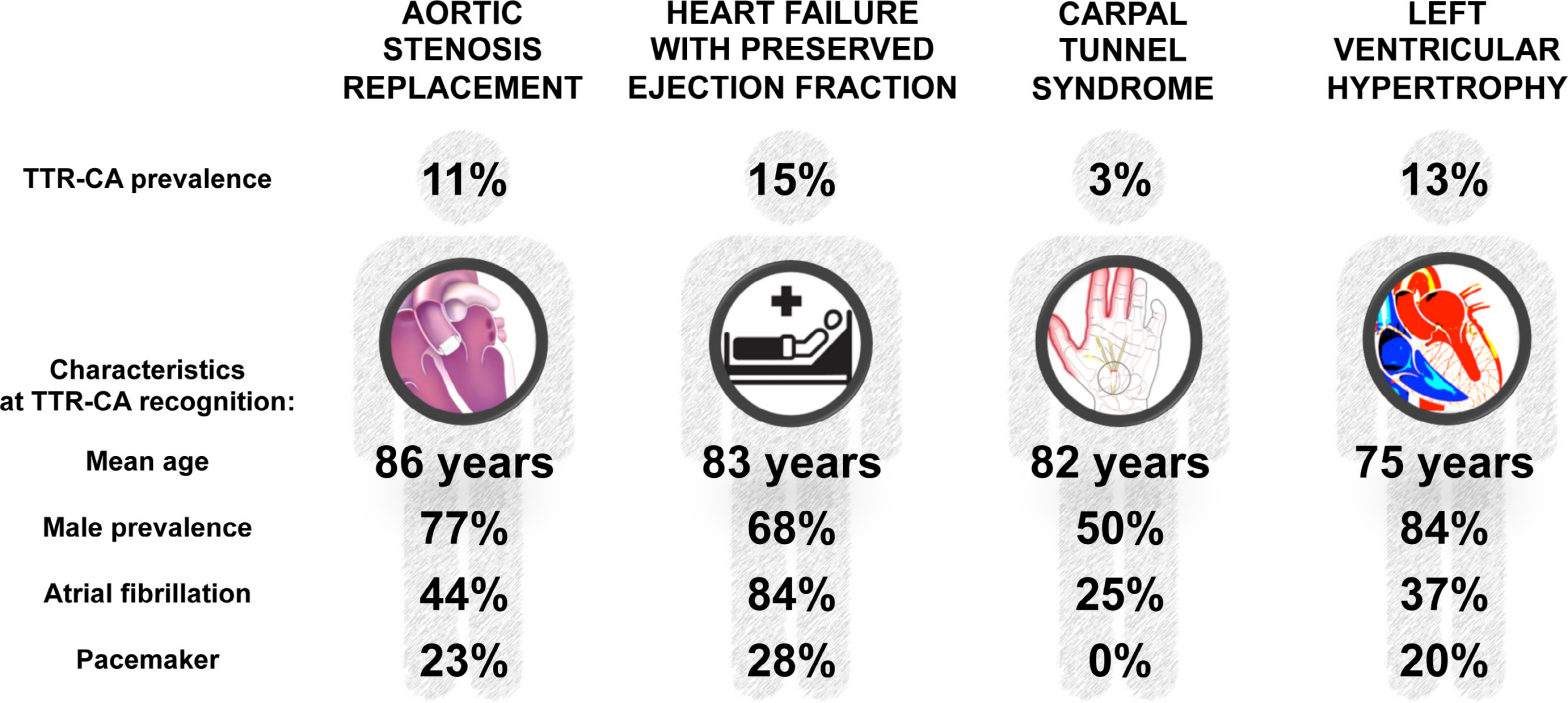
Average characteristics of TTR‐CA patients diagnosed through screening of conditions at risk using bone scintigraphy

Apart from CTS, in which TTR‐CA prevalence was 2.6%, the overall yield of screening for TTR‐CA ranged between 11.4% and 14.8%. However, characteristics of screened and diagnosed patients varied significantly across different conditions (Table 1, Figure 2). In particular, LVH/HCM patients were younger and possibly burdened by fewer comorbidities than AS and HFpEF patients at the time of TTR‐CA recognition. Moreover, a wider range of CA aetiologies (including cases of TTRv‐CA and the majority of other CA forms) was identified in the formers. On the contrary, in the AS and HFpEF settings, screening with bone scintigraphy identified almost exclusively patients affected by TTRwt‐CA (previously defined ‘senile’ TTR‐CA), with a mean age of 86 and 83 years, respectively. Surely, these differences reflect the diverse epidemiological background of each condition. However, age at TTR‐CA recognition has important implications. To be successful, a screening strategy must be cost‐effective and identify an unrecognized condition for which a specific management and/or treatment is expected to reduce morbidity and/or mortality.^222^ Among the studies included in this analysis, only AS ones assessed outcomes of CA patients, and found no significant differences in terms of mortality in CA versus non‐CA patients.^11^, ^12^, ^13^ One study found higher rate of heart failure hospitalizations only in the short term.^12^ Therefore, while a known diagnosis of TTR‐CA should not preclude the opportunity of undergoing aortic valve replacement,^23^ the value of routine screening for TTR‐CA in the overall elderly AS population remains unclear. More in general, to date only one specific disease‐modifying therapy, with the tetramer stabilizer agent tafamidis, is available for TTR‐CA treatment. Results from the ATTR‐ACT phase 3 trial highlighted that the efficacy of tafamidis was greater in patients with mild heart failure symptoms, and that its therapeutic effects were not exerted immediately, but after approximately 18 months.^24^ Thus, considering the substantial costs of tafamidis treatment^25^ and its survival benefits expected only in the long term, the overall eligibility for this therapy of the elderly TTR‐CA AS or HFpEF populations should be further evaluated and could likely be limited. Apart from age, the clinical characterization offered by the studies included in this analysis is limited. While it appears likely that elderly AS or HFpEF patients (vs. relatively younger LVH/HCM ones) have a greater burden of comorbidities, consistent data were available only for arterial hypertension, diabetes, atrial fibrillation and pacemaker implantation. A more comprehensive and multidimensional patient clinical evaluation^26^, ^27^ appears warranted to better describe TTR‐CA patients in each condition, and to guide treatment decision‐making.

Increasing the awareness towards TTR‐CA remains of critical importance in everyday clinical practice and in clinical trials.^7^, ^28^ For example, TTR‐CA patients may not tolerate standard heart failure drugs,^29^ and, if not adequately recognized, jeopardize epidemiology and results of HFpEF trials.^30^, ^31^ Thus, the results of this work should not be interpreted to question the usefulness of screening strategies aimed at refining recognition of TTR‐CA, but to highlight current gaps in the methodology and outcomes of TTR‐CA screening studies reported in the literature. Since TTR‐CA medications are supposed to be the most effective in the early phases of the disease and after a relatively long interval of treatment, we believe that screening strategies should address conditions where younger and fitter patients would likely be identified. A dedicated clinical trial (NCT 04424914) is ongoing and may offer an important insight into the real prevalence and accurate characteristics of TTR‐CA diagnosed among HFpEF patients older than 60 years and presenting LVH. In addition, a nationwide Italian survey is taking place to evaluate the prevalence of TTR‐CA among all‐comers LVH patients at echocardiography laboratories (data not published, manuscript under review). The authors are not aware of other large initiatives of this kind focused on at‐risk conditions discussed in this work. Finally, the only two previous works similar to the present one were as follows: (i) an editorial article summarizing evidences collected in AS patients with and without TTR‐CA, but without considering the significant overlap between study populations,^32^ as we did, and (ii) a brief meta‐analysis of four studies investigating the prognostic impact of TTR‐CA diagnosis in AS studies, and identifying the degree of left ventricular wall thickness as the major prognostic determinant in patients with dual pathology.^33^

## 5 CONCLUSIONS

Studies screening for TTR‐CA in specific populations considered at risk found a varying prevalence, particularly relevant in AS, HFpEF and LVH/HCM. Nevertheless, characteristics of TTR‐CA patients diagnosed in these settings were different, and those with AS and HFpEF were typically older than 80 years of age and with multiple comorbidities. Considering costs and efficacy of contemporary available disease‐modifying therapies, these results question whether these conditions represent adequate screening settings for the early recognition of TTR‐CA. Moreover, several of the screening studies included in the present work have inherent methodological limitations that may have partly hindered their accuracy and that should be addressed in future prospective studies.

## CONFLICTS OF INTEREST

All authors reported no conflicts related to the present work. M.C. received speaker and/or advisor fees in the last 2 years from Akcea Therapeutics, Menarini, Novartis, Pfizer, Sanofi e Sanofi Genzyme, Vifor Pharma and two investigator‐initiated grants from Pfizer, all outside of the scope of the present work.

## Supporting information

Supplementary Material
